# Alcohol Use and the Risk of Communicable Diseases

**DOI:** 10.3390/nu13103317

**Published:** 2021-09-23

**Authors:** Neo K. Morojele, Sheela V. Shenoi, Paul A. Shuper, Ronald Scott Braithwaite, Jürgen Rehm

**Affiliations:** 1 Department of Psychology, University of Johannesburg, Johannesburg 2006, South Africa; 2 Section of Infectious Diseases, Department of Medicine, Yale University School of Medicine, New Haven, CT 06510, USA; sheela.shenoi@yale.edu; 3 Yale Institute for Global Health, Yale University, New Haven, CT 06520, USA; 4 Centre for Addiction and Mental Health, Institute for Mental Health Policy Research and Campbell Family Mental Health Research Institute, Toronto, ON M5S 2S1, Canada; paul.shuper@camh.ca (P.A.S.); jtrehm@gmail.com (J.R.); 5 Dalla Lana School of Public Health, University of Toronto, Toronto, ON M5T 3M7, Canada; 6 Institute for Collaboration on Health, Intervention, and Policy, University of Connecticut, Storrs, CT 06269, USA; 7 Alcohol, Tobacco and Other Drug Research Unit, South African Medical Research Council, Pretoria 0001, South Africa; 8 Division of Comparative Effectiveness and Decision Science, Department of Population Health, NYU Grossman School of Medicine, New York University, New York, NY 10013, USA; Scott.Braithwaite@nyulangone.org; 9 Department of Psychiatry, University of Toronto, Toronto, ON M5T 1R8, Canada; 10 Center for Interdisciplinary Addiction Research (ZIS), Department of Psychiatry and Psychotherapy, University Medical Center Hamburg-Eppendorf (UKE), 20246 Hamburg, Germany; 11 Institute of Clinical Psychology and Psychotherapy, Technische Universität Dresden, 01187 Dresden, Germany; 12 Faculty of Medicine, Institute of Medical Science, University of Toronto, Toronto, ON M5S 1A8, Canada; 13 Program on Substance Abuse, Public Health Agency of Catalonia, 08005 Barcelona, Spain; 14 Department of International Health Projects, Institute for Leadership and Health Management, I.M. Sechenov First Moscow State Medical University (Sechenov University), 119991 Moscow, Russia

**Keywords:** alcohol, communicable diseases, infectious diseases, HIV, tuberculosis, pneumonia, severe acute respiratory syndrome coronavirus 2

## Abstract

The body of knowledge on alcohol use and communicable diseases has been growing in recent years. Using a narrative review approach, this paper discusses alcohol’s role in the acquisition of and treatment outcomes from four different communicable diseases: these include three conditions included in comparative risk assessments to date—Human Immunodeficiency Virus (HIV)/AIDS, tuberculosis (TB), and lower respiratory infections/pneumonia—as well as Severe Acute Respiratory Syndrome Coronavirus 2 (SARS-CoV-2) because of its recent and rapid ascension as a global health concern. Alcohol-attributable TB, HIV, and pneumonia combined were responsible for approximately 360,000 deaths and 13 million disability-adjusted life years lost (DALYs) in 2016, with alcohol-attributable TB deaths and DALYs predominating. There is strong evidence that alcohol is associated with increased incidence of and poorer treatment outcomes from HIV, TB, and pneumonia, via both behavioral and biological mechanisms. Preliminary studies suggest that heavy drinkers and those with alcohol use disorders are at increased risk of COVID-19 infection and severe illness. Aside from HIV research, limited research exists that can guide interventions for addressing alcohol-attributable TB and pneumonia or COVID-19. Implementation of effective individual-level interventions and alcohol control policies as a means of reducing the burden of communicable diseases is recommended.

## 1. Introduction

Alcohol consumption was recognized as a risk factor for infectious lung diseases, such as pneumonia, as early as 1785, in Benjamin Rush’s seminal work on the effects of spirits on the human body and mind [1]. However, the first global comparative risk assessment on alcohol use as a risk factor for disease burden and mortality, conducted in the last decade of the last century [2], did not include any effects of alcohol consumption on infectious disease. The impact of alcohol use on infectious disease outcomes only entered comparative risk assessments in the Global Burden of Disease Study and the World Health Organization’s (WHO) Global Status Reports after 2010 (starting with [3]; for an overview of the reasoning to include it, see [4,5]).

We can only speculate as to why the strong association between alcohol use and infectious disease was overlooked in global risk assessments for such a long time. This oversight is all the more astonishing as the association is readily apparent in research and practice, for instance, by the high prevalence of people with alcohol use disorders (AUDs) in tuberculosis treatment [6]; or by the strong associations between alcohol use and HIV/AIDS in surveys or other empirical studies [7,8,9,10,11,12]. However, these associations were not judged to be necessarily causal, even when alcohol use was related to the incidence of HIV infection [13,14]. Another likely reason is that the impact of alcohol use was indirect, via behavioral and biological pathways which were impacted by many social and other factors, making it difficult to identify alcohol use as a necessary element in a multi-component process of causation [15].

For example, the impact of alcohol on HIV/AIDS is mainly mediated by the impact of alcohol use on decision-making, resulting in riskier sexual behaviors [16] and lower adherence to virus suppression therapies [17,18,19,20], which results in higher transmission of HIV and other sexually transmitted diseases [21,22,23]. It took strong experimental methodology to ascertain that the widely recognized associations between alcohol and HIV/AIDS had substantial causal components (for more details, see [16,17]). For other infectious disease outcomes, such as tuberculosis (TB), the toxic effects of heavy alcohol consumption on the immune system render the host more susceptible to TB disease, which is again an indirect effect ([24,25]; for systematic overviews on all mechanisms, see [6,26]).

Given the plethora of multi-component causal pathways involving alcohol and infectious diseases and the complexities required to elucidate them, additional evidence is likely to continue to emerge regarding the causal impact of alcohol use on infectious diseases. For example, there is an association between alcohol and other sexually transmitted infections [27], and the causal mechanism for the impact of alcohol on HIV infection seems to also apply to these other sexually transmitted infections [5]. However, the present review will be restricted to conditions that have been included in global comparative risk assessments to date (HIV/AIDS, tuberculosis, pneumonia) with one exception, COVID-19 infection, which has been included because of its recent and rapid ascension as a global health concern, even though it occurred after the last global comparative risk assessment was performed. All sections on disease outcomes discuss both behavioral and biological risk factors and are split into sections regarding incidence (Does alcohol use cause new infections with the disease?) and impact upon the course (How does alcohol use impact the course of disease?), and all sections also discuss different dimensions of alcohol consumption, in particular, irregular and heavy drinking occasions.

## 2. Alcohol and the Risk of Human Immunodeficiency Virus (HIV) and Acquired Immune Deficiency Syndrome (AIDS)

HIV persists as a global health issue. In 2020, there were an estimated 37.6 million people living with HIV, including 1.5 million newly infected individuals and 690,000 who died from AIDS-related illnesses [28]. Alcohol has been identified as a driver of this epidemic, facilitating HIV acquisition/transmission and disease progression through both behavioral and biological means.

### 2.1. Alcohol and HIV Acquisition/Transmission

#### 2.1.1. Behavioral Mechanisms

Most HIV seroconversions result from sexual activity [29], and alcohol has been associated with a diminished likelihood of engagement in the behaviors necessary to prevent sexually based HIV acquisition/transmission. Consuming alcohol in sexual contexts can result in alcohol myopia [30], which entails an alcohol-induced constraint in cognitive capacity that causes a focus on risk-impelling cues (e.g., sexual arousal) and a disregard of risk-inhibiting cues (e.g., the prospect of HIV acquisition/transmission), thereby increasing the likelihood of condomless sex. This mechanism and corresponding alcohol–condomless sex association have been supported through a number of reviews and meta-analyses [7,8,9,10,11,12,14,31] as well as through controlled experiments that have provided evidence for the causal nature of this link [16,17,32,33,34].

More recently, HIV prevention efforts have emphasized biomedical approaches, which include HIV Pre-Exposure Prophylaxis (PrEP)—a medication taken daily by those living without HIV to prevent HIV acquisition [35,36]; and Treatment as Prevention (TasP)—which involves people living with HIV taking antiretroviral therapy (ART) to achieve viral suppression, thereby eliminating the possibility of viral transmission [21,22,23]. Despite their biomedical basis, these approaches are directly reliant on a behavior, namely adherence, which has been shown to be negatively associated with alcohol use [18,19,20,37,38,39,40,41]. A variety of underlying mechanisms for this association have been proposed, which, for the sake of conciseness, are presented below under “Alcohol and HIV Disease Progression”. It is possible that long-acting formulations of PrEP and ART may be particularly well suited for HIV prevention in alcohol users because those formulations diminish the adherence burden. This hypothesis needs to be evaluated in future research.

#### 2.1.2. Biological Mechanisms

Alcohol use can facilitate HIV acquisition/transmission by (1) decreasing host immune efficiencies among those living without HIV and (2) increasing viral replication among people living with HIV. Regarding the former, alcohol disrupts the physiology of the liver, causing a disturbance to non-specific innate and adaptive immune responses [42,43,44,45]. Both acute and chronic alcohol consumption can suppress the production of lymphocytes and cytokines [46,47,48,49,50], inhibit T-lymphocyte proliferation [51], and decrease or inhibit the production of CD4+ and CD8+ T-cells and natural killer cells [52,53], which, taken together, can result in immunodeficiency and autoimmunity, and increase host susceptibility to HIV infection [25,54,55]. These effects can be further exacerbated by liver diseases such as liver fibrosis and cirrhosis observed among those who chronically abuse alcohol [25,54,55,56,57,58,59]. Among people living with HIV, moderate to heavy alcohol consumption has been significantly associated with changes in vaginal flora, increased proinflammatory cytokines, and genital tract inflammation, which increase HIV shedding and replication, and, in turn, the likelihood of HIV transmission [60,61,62,63].

### 2.2. Alcohol Use and HIV Disease Progression

#### 2.2.1. Behavioral Mechanisms

The successful treatment of HIV, which entails achieving viral suppression to halt disease progression, relies on enacting the behaviorally underpinned steps of the HIV care continuum that include HIV testing, linkage, and retention in HIV care, and ART initiation and adherence. Alcohol use has been associated with poor outcomes at all steps of the continuum [37,64,65,66,67,68,69], and some evidence suggestive of the *causal* role of alcohol use, particularly with respect to adherence, has been yielded [17,18,19,20]. Alcohol-HIV care continuum associations can result from a range of mechanisms, including alcohol-related stigmatization that prevents alcohol users from accessing HIV testing and care [70,71], and alcohol-derived diminished cognitive functioning that poses a challenge for ongoing adherence and clinic attendance [72,73]. Among individuals who are alcohol-dependent, the syndrome of dependence may shift priorities towards obtaining and consuming alcohol and away from health, self-care, and other concerns [70]. Finally, specific to ART adherence, some alcohol-consuming people living with HIV consciously and intentionally decide not to take their doses due to factors including the possession of beliefs surrounding toxic alcohol–ART interactions [74].

#### 2.2.2. Biological Mechanisms

The role of alcohol in HIV disease progression is manifested through its effects on host liver and immunomodulation, resulting in increased activation of CD4+ T-cells and its subsequent depletion at mucosal sites [63], as well as inhibition and abnormalities of T and B lymphocytes and natural killer cells [31,75,76], all of which are necessary for the containment of HIV pathogens. Alcohol may also enhance HIV viral replication by increasing or altering the HIV-binding CXCR4 coreceptor [77,78]. Accordingly, among ART-naïve individuals, heavy drinking (vs. lower consumption) has been linked to higher CD8 cell counts and lower CD4 cell counts [79,80,81], and among those taking ART, it has been associated with reduced CD4 cell counts and higher log HIV RNA, even after controlling for adherence and age [80,82,83,84]. Relevant to this latter group, some ART medications are metabolized by the Cytochrome P450 enzyme pathway in the liver, which may be induced or inhibited by acute or chronic alcohol consumption [63,85,86]. This can affect the pharmacokinetics of some ART medications, resulting in either an increase or decrease of the available drug in plasma and causing drug toxicity or suboptimal control of the virus, respectively [63]. The effect of alcohol on ART can be further exacerbated by comorbidities, including drug dependence and Hepatitis C coinfection [17,63,87,88,89].

### 2.3. Addressing the Intersection of Alcohol Use and HIV

Alcohol use is closely intertwined with the persistent HIV epidemic. HIV prevention- and treatment-related outcomes can be improved by addressing alcohol use through behavioral [90], pharmacological [91], and policy/structural-level interventions [92,93]. Tailoring and targeting these interventions to meet the unique needs of diverse populations affected by HIV may further enhance their effectiveness and help reduce the global HIV burden [94].

## 3. Alcohol Use and the Risk of Tuberculosis

Tuberculosis (TB) is the leading cause of infectious death globally, surpassing HIV/AIDS and among the top 10 causes of death worldwide [95]. In 2019, 10 million people became ill with tuberculosis, and 1.4 million people died [95]. TB is caused by *Mycobacterium tuberculosis*, transmitted when affected individuals cough droplet nuclei containing the bacteria into the air, which is subsequently inhaled by others, causing latent infection and pulmonary and extrapulmonary disease. The WHO estimates that one-third of the global population is latently infected. Alcohol use is among the top modifiable risk factors for tuberculosis, with AUDs prevalent in 30% of patients with TB and 11.4% (9.3–13%) of TB mortality attributable to alcohol [95,96].

### 3.1. Behavioral Mechanisms

Alcohol use is well established as a risk factor for incident TB [6,97], responsible for 17% of incident TB globally [98]. Data suggest that alcohol use is associated with a 35% increased risk for developing active TB [98], while a systematic review of 21 studies demonstrated that only heavy alcohol use (defined as >40 g ethanol daily) or AUD provided a pooled risk of 3.50 (95% CI: 2.01–5.93) [6,99]. The mechanisms for the increased risk are not clearly delineated but are likely attributable to both biological and behavioral factors, the latter facilitated by close contact in crowded congregate settings [6,26]. Alcohol consumed in the context of social interactions, such as bars [6,26,99], has facilitated transmission and outbreaks of TB in institutionalized or service settings, including prisons [100] and among homeless populations [101] have been well documented.

Alcohol use is also an established risk factor for poor TB outcomes overall, including treatment failure, loss to follow up, and mortality for both drug-susceptible and drug-resistant TB [102]. This is predominantly attributed to behavioral mechanisms, notably poor adherence to TB treatment and poor retention in TB care [102,103,104]. Outcomes are worse with common comorbid conditions, including HIV, hepatitis C, substance use, and smoking [105,106,107,108]. Recognized limitations in studies assessing the relationship between alcohol and TB include poorly quantified alcohol consumption, a lack of alcohol standards across countries, and a lack of data on optimal screening for alcohol use among those receiving TB treatment.

Increased TB treatment failure and death independent of loss to follow-up suggests alcohol-related biological factors [102], with a novel study underway to gauge the role of alcohol use on outcomes controlling for adherence [109]. Interventions to reduce the impact of alcohol use on TB outcomes are scarce though emerging evidence suggests screening and intervention are feasible and promising to improve treatment completion and clinical outcomes [56,110,111,112,113,114,115,116].

Less is known about the role of alcohol in TB preventive therapy. Until recently, the only regimen available for prevention was 6–12 months of isoniazid with rare but recognized hepatic toxicity that can be exacerbated by alcohol use [117,118,119,120,121]. The balance between the benefits of preventing TB, with its individual and public health implications, against the risk of individual toxicity is currently being explored, considering the potential lower risk associated with shorter course regimens [122,123]. Data are needed to guide optimal screening and thresholds for alcohol use that halt TB preventive therapy; strategies to improve TB preventive therapy completion in the setting of alcohol use are being evaluated [124,125].

### 3.2. Biological Mechanisms

Pathophysiologically, data suggest multiple targets of alcohol use, including direct impairment of cell-mediated immunity [126], direct impact on the upper respiratory tract [127], indirect impact on adaptive immunity [128], and malnutrition [129,130], as pathways for increased susceptibility to TB.

Alcohol use complicates TB treatment for drug-susceptible and drug-resistant TB [102]. Frequent coinfection with hepatitis C and/or HIV increases the risk of hepatotoxicity [121,131,132]. Interactions between alcohol and anti-tuberculous medications are well established. Isoniazid, rifampin, and pyrazinamide, core agents of the first-line TB treatment regimen, uncommonly (~1–3%) cause hepatitis, which can be dose-related and reversible with cessation of the medications or may be due to hypersensitivity reaction [133]. Data are emerging on newer agents now available for drug-resistant TB [134]. In patients taking TB treatment who are at increased risk of hepatotoxicity, such as those with alcohol use or liver disease, closer monitoring is recommended, and non-hepatotoxic TB agents may be substituted.

### 3.3. Addressing the Intersection of Alcohol Use and TB

Alcohol use increases the risk of incident tuberculosis disease and risk of poor outcomes, primarily through behavioral mechanisms [98,99]. Data suggest alcohol use also impairs cell-mediated and adaptive immunity, though work remains to elucidate these mechanisms [126,127]. Additionally, there is a paucity of data on the thresholds for alcohol use that portend risk, implementation of screening and addressing alcohol use within TB programs, and the alcohol-related risk for latent TB and latent TB treatment. Emerging data suggest promising interventions that can improve TB outcomes.

## 4. Alcohol Use and the Risk of Lower Respiratory Infections (Pneumonia)

Pneumonia is the most important category of lower respiratory infections. Its most common type is bacterial pneumonia caused by the *Streptococcus pneumoniae*, but other forms may be viral or, rarely, caused by fungi or parasites [135]. In 2019, lower respiratory infections, the main category usually estimated in international statistics, caused about 2.5 million deaths globally (point estimate: 2,493,000; 95% confidence interval (CI): 2,268,000–2,736,000) and about 97 million disability years of life lost (DALYs; point estimate: 97,190,000; 95% CI: 84,871,000–113,083,000; all data are based on the 2019 Global Burden of Disease Study [136]. More than 80% of the lower respiratory infection deaths [136] and more than 90% of the DALYs lost were in low- and middle-income countries (LMIC) [136], with a clear gradient in age-adjusted rates by wealth: the higher the economic wealth, the lower the rate of lower respiratory infections. In total, 3.2% (95% CI: 1.6–6.0%) of the deaths and 1.8% of the DALYs (95% CI: (1.0–3.3%)) due to lower respiratory infections were attributable to alcohol, meaning they would not have occurred in a world without alcohol [137].

Alcohol use both impacts the etiology and the course of lower respiratory infections, most importantly in community-acquired infections. As with most infections, lower respiratory infections are more highly prevalent in crowded environments often inhabited by poor people. Additionally, within countries, pneumonia is associated with socioeconomic status, an indicator of wealth: the higher the socioeconomic status, the lower the prevalence of lower respiratory infections [138]. Alcohol contributes to these inequalities ([139,140]), especially via heavy drinking occasions [141]. Of course, factors other than crowding and alcohol use, which are associated with wealth at the individual and societal levels, also contribute to lower respiratory infection rates, such as tobacco smoking, undernutrition, indoor air pollution, and insufficient access to health care [96]. Most of these risk factors are known to interact with alcohol use [142].

The main impact of alcohol use on lower respiratory infections seems to be via the innate and the adaptive immune system [24,53,143,144,145]. There are a number of pathways leading to the weakening of various aspects of the immune system, with the key immune cells involved in combating pulmonary conditions being neutrophils, lymphocytes, alveolar macrophages, and the cells responsible for innate immune responses [44,55,145,146,147,148,149]. In addition, alcohol use is causally linked to more than 200 disease and injury outcomes (such as various types of cancer, stroke, liver cirrhosis, or traffic injury) which weaken the immune system and increase the risk for lower respiratory infections [138].

Although a number of studies on pathways have been conducted among people with AUDs, two dose–response meta-analyses found an almost linearly increasing risk with increasing average consumption of alcohol [150,151]. These two meta-analyses estimated that an average increase of one drink per day was associated with an increased risk of 8% (95% CI 6–9%) and 6% (95% CI 1–11%). As people with AUDs tend to have the highest average consumption [152], risk for lower respiratory infections is highest in this group. For, instance, in a cohort study of more than 12 million French hospital patients, the relative risk for hospitalization for pneumococcal pneumonia in patients with an AUD was 3.71 (95% 3.60–3.83; [153]). Other studies have found similar and higher risks [154,155,156].

The same mechanisms which lead to the incidence of lower respiratory infections also worsen its course. Clearly, the living conditions and behaviors, as well as alcohol-induced compromised immunity, hinder the healing process for people with lower respiratory infections [157]. Abstinence or at least an absence of heavy drinking occasions should thus be the norm during such infections, bearing in mind that for some people with AUD, abruptly abstaining may lead to alcohol withdrawal syndrome, which in itself may have severely negative effects [158].

## 5. Alcohol Use and the Risk of COVID-19

The ongoing global pandemic of coronavirus disease 2019 (COVID-19) is caused by the severe acute respiratory syndrome coronavirus 2 (SARS-CoV-2). Following the first cases in China late in 2019, which soon spread to other countries, the WHO declared it a Public Health Emergency of International Concern on 30 January 2020, and later—based on more than 118,000 cases in 114 countries and 4291 deaths—a pandemic on 11 March 2020 [159]. To date, on 10 July 2021, the estimates are that there have been more than 187 million COVID-19 infections and more than 4 million deaths [160], which are widely considered to be conservative estimates, as only direct cases with ascertainment are included (for a total estimate, see [161]). Alcohol use may play a role in both the incidence and the course of the disease [162], with both behavioral and biological pathways.

### 5.1. Behavioral Pathways

COVID-19–alcohol behavioral pathways hinge on the social drift hypotheses—the phenomenon that alcohol problems, especially in heavy drinkers and people with AUDs, are associated with long-term negative effects on the place of residence, involving an elevated likelihood of moving into or remaining in disadvantaged neighborhoods [163,164]. These environments hinder physical distancing and have been established as risk factors for COVID-19 infections and poor outcomes [165]. Independent of characteristics of disadvantaged neighborhoods, alcohol consumption has been shown to narrow physical distancing [166].

### 5.2. Biological Pathways

COVID-19 has only recently emerged as a pathogen, so there is less comprehensive and systematic knowledge about its relationship with alcohol use than there is for more established pathogens. The pathophysiology of COVID-19 is complex [167] but can be conceptually simplified as:

(1) COVID-19 travels from the upper respiratory tract (highest transmission risk) to the lower respiratory tract (highest disease risk), causing pneumonia;
(2) COVID-19 initiates innate and adaptive immune responses that are often maladaptive, leading to ineffective pathogen eradication combined with inflammation that causes host tissue damage;
(3) Damage is concentrated not at the alveolus (i.e., the interface of air–blood oxygen exchange), as is typical of pneumonia, but instead at epithelial cells (i.e., cells lining the lower respiratory tract) and endothelial cells (i.e., cells lining blood vessels);
(4) Endothelial damage occurs not only in the lungs but also systematically, leading to vasculitis (i.e., damaged small blood vessels) and thrombosis (i.e., blood clots), potentially causing multi-organ failure.

Accordingly, alcohol use may impact COVID-19 by facilitating some or all of these steps. Heavy alcohol consumption is a well-known risk factor for aspiration pneumonia, so it is likely that alcohol, if consumed heavily, leads to increased aspiration of the upper respiratory tract COVID-19 to the lower respiratory tract.

Heavy alcohol use weakens the innate and adaptive immune systems [168,169]. The processes have been described in other sections and are summarised in [126,128]. A recent network meta-analysis [170] explored the potential effects of alcohol use on inflammation, based on the fact that many COVID-19 patients present with fever in the early phase, with some progressing to a hyperinflammatory phase. This network meta-analysis demonstrated that alcohol exposure might augment COVID-19-induced inflammation by altering the activity of key inflammatory mediators (augmenting inflammatory effects and inhibiting the activity of anti-inflammatory mediators, including the glucocorticoid receptor). Finally, a large study showed genetically informative putative causal effects of alcohol use on worsening the course of COVID-19 [171]. However, the last study has not undergone peer review as of yet.

Finally, chronic heavy use of alcohol leads to frailty, arterial hypertension, and liver and other organ damage, rendering people more susceptible to COVID-related complications. Susceptibility to COVID is also enhanced if alcohol leads to obesity or co-occurring infectious diseases or arterial hypertension [172,173,174,175]. Pathways involving obesity, a known independent risk factor for COVID-19 [176], have received research interest, both from theoretical [177] and empirical perspectives [173].

### 5.3. Association with Alcohol Use or Heavy Alcohol Use/AUDs

Studies on the association between alcohol use and the incidence and severity of COVID-19 have yielded mixed results. While some studies have found associations, in particular for heavy drinkers [178,179,180] or people with AUDs [181], other studies have demonstrated that alcohol use per se was not necessarily associated with the incidence of COVID-19 or with a more severe course of the disease [182,183,184]. This is in line with the postulated pathways described above, which mainly report effects for heavy drinking and/or in people with AUDs (see also [185,186,187]).

## 6. Interventions for Preventing Transmission and Improving Treatment Outcomes of Alcohol-Attributable Diseases

The evidence reviewed above suggests that alcohol is a clear risk factor for the incidence of and poor treatment outcomes from HIV, TB, and pneumonia, with the evidence regarding its effects on COVID-19 still emerging. Alcohol-attributable TB, HIV, and pneumonia combined were responsible for approximately 360,000 deaths and 14 million DALYs in 2016 (Table 1), and alcohol-attributable TB deaths and DALYs far exceeded alcohol-attributable lower respiratory infection and HIV deaths and DALYs [137]. Given the observed role of alcohol use in these diseases, reductions in alcohol consumption should lead to reduced incidence of and improved disease outcomes, including fewer deaths, among those with these illnesses. Feasible and effective alcohol-reduction interventions must be prioritized, but how best to intervene has not been fully delineated. We discuss individual-level interventions followed by structural interventions (or alcohol control measures) that may prevent transmission and improve treatment outcomes of alcohol-attributable communicable diseases.

### 6.1. Reducing the Incidence of Communicable Diseases

Individual-level approaches focusing on alcohol reduction in order to reduce the incidence of pneumonia and TB are relatively rare, whereas more studies focused on alcohol use reduction for preventing HIV transmission have been conducted. Within a systematic review of studies of interventions for reducing the incidence of TB, no studies that examined alcohol-reduction interventions for reducing the incidence of TB were found [188].

Similarly, despite the role of alcohol use in increasing the risk of pneumonia acquisition, alcohol use reduction seems to be missing as part of a number of texts providing recommendations for the prevention of pneumonia (e.g., [189,190]). On the other hand, one of the main preventative measures for pneumonia is vaccination, and commentators have recommended vaccinating individuals with an AUD in order to prevent (re-)infection with pneumonia [158]. Others (e.g., [191]) have suggested that clinicians should identify individuals who are at high risk of developing pneumonia as potential candidates for pneumonia vaccinations due to their possession of risk factors, including alcohol use, smoking, older age, and lower socioeconomic status, among a few others [191].

In terms of HIV, a number of systematic reviews of alcohol–HIV reduction interventions [8,192,193], mostly conducted in clinic or treatment settings, have shown that behavioral interventions can reduce alcohol use in sexual contexts and alcohol consumption among individuals at risk of alcohol-related HIV acquisition. A systematic review [8] of alcohol–HIV interventions targeting both alcohol and sexual risk behavior reduction among STI clinic and substance use treatment patients in Russia showed evidence of effectiveness in increasing condom use. Interventions in other settings, such as bars and communities, may also be ideal and feasible (e.g., [194,195,196]) but have yielded mixed results [194,195]. Secondary prevention, which entails TasP (discussed below), with high adherence to ART to bring about viral suppression, is particularly important yet problematic in people living with HIV who drink alcohol [94].

### 6.2. Improving Treatment Outcomes

Since alcohol use complicates the treatment of many communicable diseases, integration of alcohol use reduction counseling or screening and brief interventions into TB [197], HIV [94], or pneumonia [150] treatment services has been recommended. Similarly, screening for TB [197] or HIV among people with AUDs has also been recommended, as has the co-location of services [94]. However, the evidence base regarding the effectiveness of such approaches for all communicable disease categories of interest in the current report is fairly limited.

A few primary studies that have evaluated the efficacy of individual-level alcohol reduction interventions for improving TB treatment outcomes [56,114,116,198] have yielded disappointing results. In Russia, Shin et al. [198] found no differences between the TB and alcohol use outcomes of new TB patients with AUDs who received: (1) a brief counseling intervention (BCI) and treatment as usual; (2) naltrexone combined with brief behavioral compliance enhancement counseling (BBCET) (naltrexone adherence counseling); (3) BCI and naltrexone with BBCET and treatment as usual; and (4) treatment as usual—referral to a narcologist (namely, an addiction psychiatrist in the Russian system). One sub-group analysis revealed that among those with previous quit attempts (*n* = 111), the TB treatment outcome was better for the naltrexone group (92.3%) compared with the non-naltrexone group (75.9%). In a cluster RCT in South Africa, Peltzer et al. [116] found no effect for a two-session screening and brief intervention on TB and alcohol use outcomes among new TB patients who had Alcohol Use Disorder Identification Test (AUDIT) scores of ≥7 if they were women and ≥8 if they were men. More research on individual-level alcohol-reduction interventions among patients on TB treatment is needed.

Several recommendations regarding the treatment of patients with pneumonia who drink alcohol or have AUDs have been put forward. These include preventing further bouts of pneumonia by providing alcohol counseling [151] and pneumococcal vaccination [158]. Screening and brief interventions for AUDs among all patients undergoing treatment for pneumonia have also been recommended so that the clinician can be well informed about their patients’ alcohol use and manage their pneumonia accordingly [150]. Assessment for potential alcohol withdrawal syndrome that may occur as a result of abstinence is also recommended as it can have serious and even fatal consequences if not managed appropriately [158]. Providing guidelines on screening for the risk of AUDs and alcohol withdrawal syndrome to TB treatment providers has also been recommended [199].

Efforts to improve treatment outcomes and improve secondary prevention for people living with HIV who drink alcohol require emphasizing linkage and retention in care, ART initiation, ART adherence, viral suppression, and condom use [94]. A recently published high-quality systematic review and meta-analysis involving 21 studies and 8461 people living with HIV, 69% of whom were on ART, has indicated (contrary to other findings, [200,201,202]) that individual-level behavioral interventions were effective in reducing the quantity and heavy consumption (but not alcohol use or alcohol use frequency), increasing condom use (but not affecting the number of sexual partners or a composite index of sexual risk), reducing viral load, and increasing ART adherence [90]. Interventions in which participants were recruited from clinics were most likely to be effective. As supplements to such interventions, additional approaches that have been recommended include the use of technology to deliver interventions, use of ultra-brief interventions, prevention of increased alcohol consumption or the development of AUDs, a focus on aging populations, addressing psychosocial comorbidities, and improving accessibility and convenience of HIV care [94]. Occasionally, health workers have stigmatizing attitudes or inadequate knowledge that can lead to inadvertent ART nonadherence among their patients [203]. Very clear guidelines are needed to enable health workers to provide appropriate and consistent messaging [204].

Pharmacological interventions for reducing alcohol use and improving treatment outcomes may be especially appropriate for those with communicable diseases [91]. Farhadian et al.’s systematic review, including seven studies, provided some evidence of naltrexone’s effectiveness in reducing alcohol consumption and HIV viral load, but it did not affect ART adherence, CD4 cell count, or disease severity. However, as discussed above, a study in which naltrexone was used in combination with naltrexone adherence counseling, and in another group also behavioral counseling to reduce drinking and improve TB treatment outcomes among TB patients, did not yield positive results [198].

### 6.3. Alcohol Control Measures

Alcohol control policies that are aligned with the three alcohol “best buys”—increasing excise tax, bans or restrictions on alcohol advertising, and restricting the availability of alcohol [205]—are most effective for reducing population-level alcohol use. These measures can be expected to be effective for reducing the incidence of and morbidity and mortality due to alcohol-attributable TB, pneumonia, and HIV. There is some evidence that may provide support for such effects. For example, a study in the United States showed that longer sales hours at the state/district level were associated with high-risk sexual behaviors [92], which are associated with HIV transmission. However, implementation of effective alcohol control policies such as the best buys is relatively low around the world, particularly in lower- and middle-income countries [206,207,208,209], many of which have the highest disease burden with respect to many communicable diseases [136]. Implementation and enforcement of effective alcohol control policies as a means of reducing the burden of communicable diseases is recommended.

## 7. Discussion/Conclusions

Alcohol use is a clear risk factor for the incidence of and poor treatment outcomes from HIV, TB, and pneumonia. Emerging evidence suggests that heavy and chronic alcohol use is associated with an increased risk of acquisition of COVID-19 and more severe disease once infected, while evidence regarding the role of alcohol use per se in COVID-19 is mixed.

**Figure 1:**
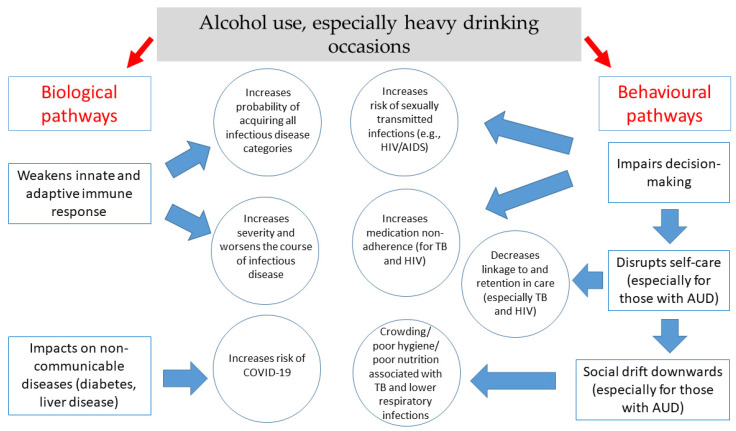
Key biological and behavioral mechanisms through which alcohol use is associated with communicable diseases.

Alcohol’s role in communicable diseases can be explained by both behavioral and biological mechanisms (Figure 1). Alcohol use increases susceptibility to infectious diseases through several immunologic mechanisms. Chronic or irregular heavy drinking leads to increased susceptibility to viral and bacterial infections, including mycobacterial infections and decreased response to vaccination. Chronic heavy drinking stimulates inflammation yet impairs neutrophil function in the innate (immediate) immune response and leads to loss of T cells and B cells in the adaptive (delayed or humoral) response. In contrast, moderate alcohol consumption seems to strengthen the response to infection, though the exact mechanisms of alcohol’s mixed effects on the immune system, particularly on the adaptive immune response, remain under investigation [126,128]. In terms of behavioral mechanisms, most of alcohol’s effects on disease acquisition result from impaired decision making (or impaired control), which gives rise to increased risk behaviors such as condomless sex. Furthermore, alcohol consumption is negatively associated with linkage to and retention in care and with medication adherence (particularly for HIV and TB treatment).

There has been limited research that has identified effective interventions for addressing alcohol-attributable TB and pneumonia, suggesting an urgent need for research in these areas, while several effective interventions to address alcohol-attributable HIV infection have been determined. Implementation of effective individual-level interventions, as well as alcohol control measures as a means of reducing the burden of communicable diseases, are recommended.

## Figures and Tables

**Table 1:** Alcohol-attributable communicable diseases: 2016 estimates [137].

	Deaths (Thousands)	DALYs (Millions)
Tuberculosis	236.3 (74.6–456.6)	9.9 (3.2–18.6)
HIV/AIDS	30.4 (22.8–56.7)	1.7 (1.2–3.1)
Lower respiratory infections	95.2 (48.5–177.6)	2.3 (1.3–4.3)
